# Supplementary material for: EpiCure (Epithelial Curation): a versatile and handy tool for curation of epithelial segmentation
Source: bioRxiv. 2026 Mar 27:2026.03.27.714683. Preprint. [Version 1] doi: 10.64898/2026.03.27.714683 (PMC13041987; doi:10.64898/2026.03.27.714683)
Supplement: Supplement 7 [file NIHPP2026.03.27.714683v1-supplement-7.pdf]

Figure Sup 4

A False event detections due to segmentation errors

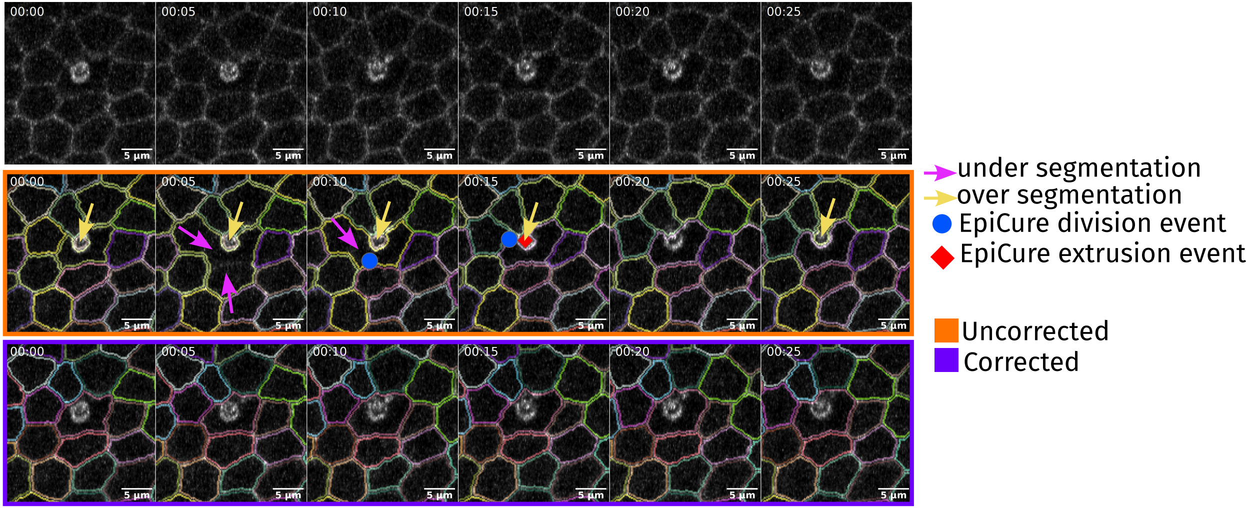

B

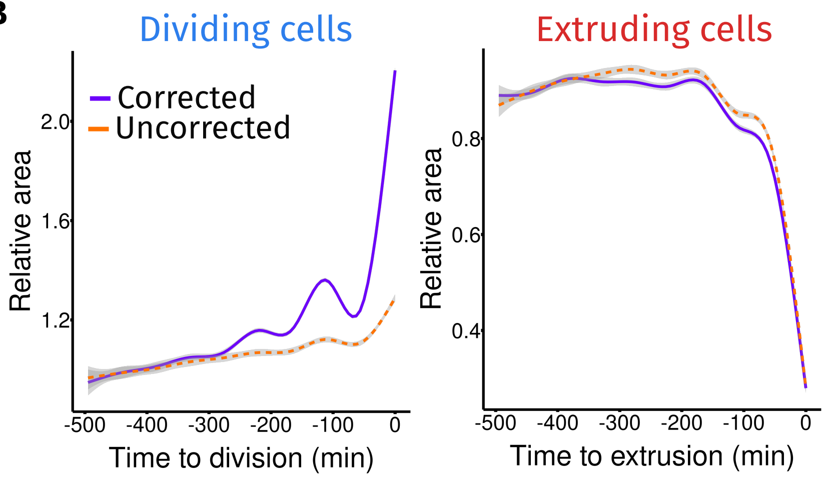

bioRxiv preprint doi: https://doi.org/10.64898/2026.03.27.714683; this version posted March 27, 2026. The copyright holder for this preprint (which was not certified by peer review) is the author/funder, who has granted bioRxiv a license to display the preprint in perpetuity. It is made available under aCC-BY 4.0 International license.

Figure Sup 4: Impact of segmentation errors on cellular events detection

(A) False event detections created by segmentation errors. Top: crop of the raw movie on a zone of interest. Middle: Segmented movie without correction: under segmentation is visible in three parts of the movie, where junction signal is faint (pink arrows). Over segmentation is visible in 5 frames, due to the SO cells (yellow arrows). These errors generate wrong tracking and thus false events (division and extrusion) detection. Bottom: Corrected segmentation. No events are detected anymore in this cropped movie.

(B) Impact of segmentation correction on events temporal distribution. Temporal evolution of the cell area relative to the area of its neighbours, for dividing cells (left) or extruding cells (right). Relative cell areas are aligned according to the time of cytokinesis termination (left) or closure of apical area (right) corresponding to time 0. Orange lines are for uncorrected movies, and magenta lines after segmentation correction. Grey area is the confidence interval at 0.95.

Figure Sup 5

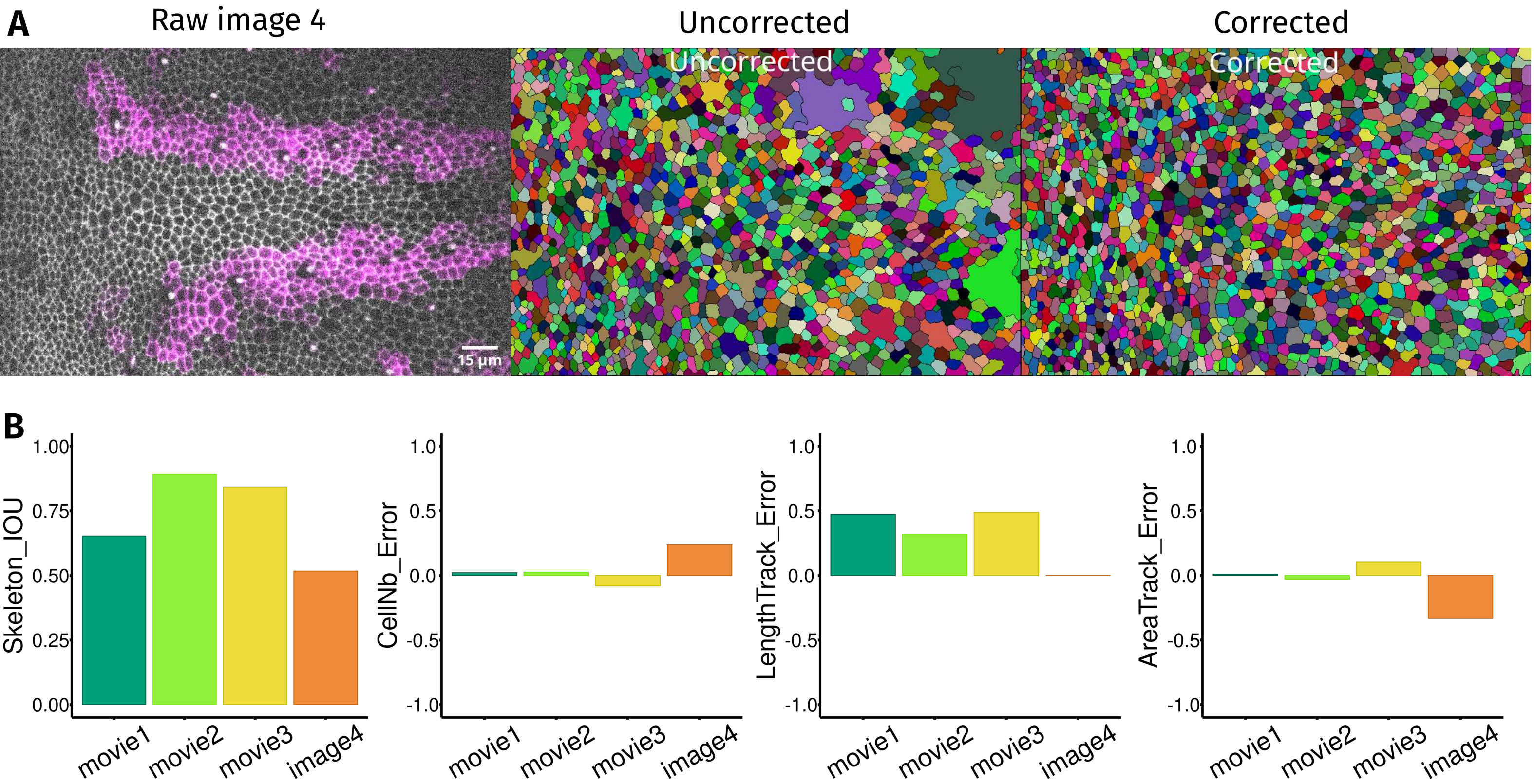
A
Raw image 4
Uncorrected
Corrected
15 µm
Uncorrected
Corrected
B
Skeleton_IOU
1.00
0.75
0.50
0.25
0.00
CellNb_Error
LengthTrack_Error
AreaTrack_Error
1.0
0.5
0.0
-0.5
-1.0
movie1
movie2
movie3
image4

bioRxiv preprint doi: https://doi.org/10.64898/2026.03.27.714683; this version posted March 27, 2026. The copyright holder for this preprint (which was not certified by peer review) is the author/funder, who has granted bioRxiv a license to display the preprint in perpetuity. It is made available under aCC-BY 4.0 International license.

Figure Sup 5: Segmentation error estimation for the four biological model systems

(A) Raw image of a *Drosophila* notum expressing E-cad::GFP (grey) with cells expressing RFP in the stripe-Gal4 domain (magenta) (left), segmentation before (middle) and after (right) correction of the 4th example of Figure 5, called “Image4”. Scale bar is 15μm.

(B) Estimation of the segmentation accuracy (the skeleton binary files before and after correction are compared, measuring the intersection-over-union (IOU) of the binaries), cell number error, length track error and area track error in the four model systems (see Figure 5). Error is defined as the relative difference between the measure after and before correction as: (after_correction – before_correction)/after_correction.

Movie legends

Movie S1: EpiCure interface and display options. The full Napari interface is recorded while changing the display options of EpiCure.

Movie S2: Movie of the *Drosophila* pupal notum from 20 to 32 hours after pupal formation (left), with cell contours marked with E-Cad::GFP (grey). Cell segmentation (one color=one cell) before (middle) and after (right) correction. Scale bar=30μm.

Movie S3: Movie of the *Drosophila* pupal notum from 20 to 32 hours after pupal formation (left), with cell contours marked with E-Cad::GFP (grey). Detection of cellular events and marking of cell fate: cells that will divide later (blue) or extrude (red) for uncorrected segmentation (left) and after segmentation curation (right). The SOs (Sensory Organs) are shown in green. Scale bar=30μm.

Movie S4: Movie of quail gastrula extraembryonic tissue showing cytoplasmic membrane GFP (grey, left) with segmentation before (middle) and after (right) correction. Scale bar=15μm.

Movie S5: Movie of *Drosophila* abdominal histoblasts with contours marked with E-cad::GFP (grey, left) with segmentation before (middle) and after (right) correction. Scale bar=15μm.

Movie S6: Movie of the adult neural stem cell layer of the zebragish telencephalon with contour marked with gpap:Zo1::mKate2 (grey) and deltaA:eGFP (orange) (left) with segmentation before (middle) and after (right) correction. Scale bar=15μm.
